# The Cholinergic Pathways in Inflammation: A Potential Pharmacotherapeutic Target for COPD

**DOI:** 10.3389/fphar.2018.01426

**Published:** 2018-12-03

**Authors:** Mitsuhiro Yamada, Masakazu Ichinose

**Affiliations:** Department of Respiratory Medicine, Tohoku University Graduate School of Medicine, Sendai, Japan

**Keywords:** acetylcholine, cholinergic anti-inflammatory pathway, chronic obstructive pulmonary disease, neuroimmune interactions, α7 nicotinic acetylcholine receptor

## Abstract

In COPD, the activity of the cholinergic system is increased, which is one of the reasons for the airflow limitation caused by the contraction of airway smooth muscles. Therefore, blocking the contractive actions with anticholinergics is a useful therapeutic intervention to reduce the airflow limitation. In addition to the effects of bronchoconstriction and mucus secretion, accumulating evidence from animal models of COPD suggest acetylcholine has a role in inflammation. Experiments using muscarinic M_3_-receptor deficient mice or M_3_ selective antagonists revealed that M_3_-receptors on parenchymal cells, but not on hematopoietic cells, are involved in the pro-inflammatory effect of acetylcholine. Recently, combinations of long-acting β2 adrenergic agonists (LABAs) and long-acting muscarinic antagonists (LAMAs) have become available for COPD treatment. These dual long-acting bronchodilators may have synergistic anti-inflammatory effects because stimulation of β2 adrenergic receptors induces inhibitory effects in inflammatory cells via a different signaling pathway from that by antagonizing M_3_-receptor, though these anti-inflammatory effects have not been clearly demonstrated in COPD patients. In contrast to the pro-inflammatory effects by ACh via muscarinic receptors, it has been demonstrated that the cholinergic anti-inflammatory pathway, which involves the parasympathetic nervous systems, regulates excessive inflammatory responses to protect organs during tissue injury and infection. Stimulation of acetylcholine via the α7 nicotinic acetylcholine receptor (α7nAChR) exerts inhibitory effects on leukocytes including macrophages and type 2 innate lymphoid cells. Although it remains unclear whether the inhibitory effects of acetylcholine via α7nAChR in inflammatory cells can regulate inflammation in COPD, neuroimmune interactions including the cholinergic anti-inflammatory pathway might serve as potential therapeutic targets.

## Introduction

Acetylcholine (ACh), a neurotransmitter released from nerve terminals of postganglionic parasympathetic neurons, promote the contraction of airway smooth muscles as well as mucus secretion via muscarinic type 3 (M_3_) receptor in the airways (Roffel et al., 1988; Mak and Barnes, 1990). It has been also revealed that non-neuronal cells including airway epithelial cells can synthesize and release ACh, which may induce biological functions in an autocrine or paracrine manner (Proskocil et al., 2004; Kummer and Krasteva-Christ, 2014). ACh is significantly involved in the pathogenesis of COPD because the activity of the cholinergic system is increased in this disease augmenting the airflow limitation and hypersecretion (Gross and Skorodin, 1984). In addition to these roles in the pathogenesis of COPD, accumulating evidence has revealed that ACh can regulate inflammatory responses. In this review, we summarize recent literature on the roles of cholinergic pathways in inflammation, especially focusing on: (1) pro-inflammatory effects of ACh through mAChRs; (2) possible benefits of the combinations of long-acting β2 adrenergic agonists (LABAs) and long-acting muscarinic antagonists (LAMAs) in terms of regulating inflammation; (3) anti-inflammatory effects of ACh via the α7 nicotinic acetylcholine receptor (α7nAChR); (4) recent advances in clarifying neuroimmune interactions that regulating innate lymphoid cells (ILCs), which could lead to potential pharmacotherapeutic targets for COPD.

## Pro-Inflammatory Actions of ACh Through Muscarinic ACh Receptors in Airways

In addition to the role of ACh in neurotransmission, the activation of muscles and mucus secretion, accumulating evidence from animal studies using muscarinic antagonists or subtype-specific deficient mice has suggested that ACh can augment airway inflammation through muscarinic ACh receptors. Tiotropium, one of the LAMAs with higher selectivity for M_3_ receptors, inhibited pulmonary neutrophilic inflammation and the release of leukotriene B4 (LTB4), interleukin-6 (IL-6), keratinocyte-derived chemokine(KC; IL-8 in humans), monocyte chemotactic protein-1 (MCP-1), macrophage inflammatory protein-1alpha and -2 (MIP-1α and MIP-2), and tumor necrosis factor alpha (TNF-α) in a mouse COPD model induced by cigarette smoke (Wollin and Pieper, 2010). Tiotropium also attenuated neutrophil infiltration and the elevation of IL-6 and TNF-α in cigarette smoke and virus-exposed mice (Bucher et al., 2016). Glycopyrronium, another M_3_-selective LAMA, attenuated the accumulation of neutrophils and macrophages and the elevation of inflammatory cytokines and chemokines induced by both acute and subchronic cigarette smoke exposure (Shen et al., 2014; Hsiao et al., 2018). Aclidinium bromide, another M_3_-selective LAMA, also attenuated neutrophilic infiltration in the alveolar septa in a cigarette smoke–exposed guinea pig model of COPD (Dominguez-Fandos et al., 2014). Although these LAMAs are kinetically selective for M_3_ receptors due to their dissociation time from M_3_ being longer than that from M_2_ or M_1_, the binding affinity of these agents for M_1_, M_2_, and M_3_ are similar and have limited selectivity (Casarosa et al., 2009), meaning that it is still unclear which muscarinic receptor subtype was critical for the pro-inflammatory effects in a cigarette smoke-exposed animal model of COPD. To answer this question, gene-targeting in mice for each subtype of muscarinic receptor was employed. M_3_ receptor-deficient mice showed a decrease in both neutrophil accumulation in the lungs and the release of KC, a neutrophil chemotactic factor, in an acute cigarette smoke-exposed mouse model (Kistemaker et al., 2013). In contrast to M_3_ receptor-deficient mice, M_2_ receptor-deficient mice showed an increase in both neutrophil infiltration in the airspaces and the release of KC after exposure to cigarette smoke (Kistemaker et al., 2013). Because the M_2_ receptor is located on the pre-junctions of pre- and post-ganglionic pulmonary parasympathetic nerves and acts as a feedback inhibitory receptor limiting acetylcholine release (Fryer and Maclagan, 1984; Faulkner et al., 1986; Minette and Barnes, 1988), the levels of ACh are increased in the airway of M_2_ receptor-deficient mice causing an enhancement of neutrophilic inflammation via the M_3_ receptor. Mice deficient in the M_1_ receptor, which is expressed on parasympathetic ganglia and mediate neurotransmission, also shows a remarkable enhancement of neutrophil inflammation after cigarette smoke-exposure (Kistemaker et al., 2013). This seems to be paradoxical because a M_1_ receptor deficiency would cause a reduction of acetylcholine in the airway, resulting the reduction of inflammation. Because M_1_ receptors are also expressed on airway epithelial cells and regulate electrolyte and water secretion (Ishihara et al., 1992), a hypothesis explaining this paradox is that the clearance of smoke particles is impaired in M_1_ deficient mice. *In vitro* cell culture experiments also supported the pro-inflammatory actions of ACh thorough muscarinic receptors on airway parenchymal cells and inflammatory cells (Table 1). Because M_3_ receptors are expressed in various types of cells including parenchymal cells and hematopoietic cells in airways (Table 1), the question has arisen whether the pro-inflammatory effects of ACh via M_3_ receptors are mediated mainly by parenchymal cells or inflammatory cells. To answer this question, bone marrow chimeric mice were established. This investigation revealed that the accumulation of neutrophils in the airways was inhibited in M_3_ receptor-deficient mice with wild-type bone marrow, whereas wild-type mice with M_3_ receptor-deficient bone marrow showed a comparable increase in neutrophil accumulation, suggesting that M3 receptors of the airway parenchymal cells are primarily involved in the pro-inflammatory effects of ACh during cigarette smoke-induced inflammation (Kistemaker et al., 2015b).

**Table 1:** Pro-inflammatory effects of ACh on parenchymal and leukocytes via muscarinic receptors.

Type of cells	Type of receptors	Actions	Reference
**Parenchymal cells**			
Epithelial cells	M_1,_ M_2_, M_3_	Inducing the release of IL-8	Profita et al., 2008
		Stimulating the release of LTB_4_	Profita et al., 2011
		Augmenting IL-17A-induced release of IL-8	Anzalone et al., 2016
Fibroblasts	M_1,_ M_2_, M_3_	Augmenting IL-1β-induced release of IL-6 and IL-8	Costa et al., 2014
Smooth muscle cells	M_2_, M_3_	Stimulating the release of IL-6 and IL-8	Gosens et al., 2009
**Leukocytes**			
Macrophages	M_1,_ M_2_, M_3_, M_4,_ M_5_	Stimulating the release of LTB_4_	Sato et al., 1998; Buhling et al., 2007; Koarai et al., 2012
Neutrophils	M_2_, M_3_, M_4,_ M_5_	Stimulating the release of IL-8	Milara et al., 2016
		Augmenting inflammatory mediator and matrix metalloproteinase release induced by LPS and cigarette smoke extract	Milara et al., 2016
Mast cell	M_1_, M_3_, M_4,_ M_5_	Inhibiting the release of histamine	Reinheimer et al., 2000
Lymphocytes	M_1_, M_2_, M_3_, M_4,_ M_5_	Augmenting the production of antibodies and cytokines	Fujii et al., 2007


Although there is accumulating evidence for a significant role of ACh as a pro-inflammatory factor in COPD as well as clinical evidence showing that LAMAs decrease exacerbations of COPD, stronger evidence for its pro-inflammatory role in COPD patients is still limited. Treatment with tiotropium in COPD patients decreased the annual rate of exacerbations. However, the amount of IL-6 and myeloperoxidase in sputum did not decrease. Moreover, the concentration of IL-8 and MMP-9 increased in the sputum of the group treated with tiotropium (Powrie et al., 2007; Perng et al., 2009). Tiotropium treatment also did not significantly decrease IL-6 or C-reactive protein in the serum (Powrie et al., 2007). This is possibly due to the fact that tiotropium decreased the amount of mucus secretion, causing a higher concentration of the mediators in sputum. It has been reported that targeted lung denervation of parasympathetic nerves in patients with COPD induces a reduction of neutrophil accumulation and inflammatory mediators including IL-8 (Kistemaker et al., 2015a). This is the first direct evidence showing that ACh acts a pro-inflammatory factor in actual COPD patients. Further investigations including histological evaluations by bronchial biopsy will be needed to elucidate whether antimuscarinic drugs actually effect inflammation in COPD patients.

## Possible Benefits of the Combinations of LABAs and LAMAs in Terms of Regulating Inflammation During COPD

**FIGURE 1:**
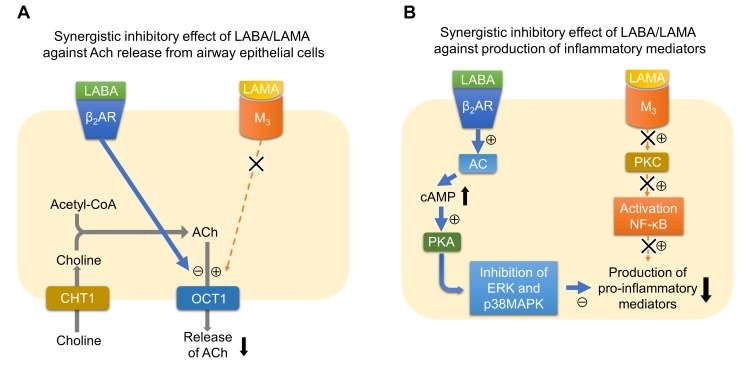
Possible mechanisms of synergistic actions of the LABA/LAMA combination against inflammation. **(A)** Synergistic inhibitory effect of LABA/LAMA against ACh release from airway epithelial cells. Signaling via β_2_AR inhibits the function of OCT1, a transporter for the release of acetylcholine from airway epithelial cells, whereas signaling via M_3_ possibly upregulates the function of OCT1. Therefore, the LABA/LAMA combination synergistically inhibits the release of ACh. **(B)** Synergistic inhibitory effect of LABA/LAMA against the production of inflammatory mediators. Signaling via β_2_AR activates AC-cAMP-PKA signaling pathway. PKA inhibits ERK and p38MAPK pathways. In contrast, signaling via M_3_ activates PKC, which results in the activation of NF-κB. The combination of LABA and LAMA therefore possibly inhibits the production of pro-inflammatory mediators synergistically via inhibiting both MAPK and NF-κB pathways. ACh, acetylcholine; AC, adenylate cyclase; β_2_AR, beta-2 adrenergic receptor; cAMP, cyclic adenosine monophosphate; CHT1, high-affinity choline transporter 1; ERK, extracellular signal-regulated kinase; LABA, long-acting β_2_ agonist; LAMA, long-acting muscarinic antagonist; MAPK, mitogen-activated protein kinase; M_3_, acetylcholine muscarinic M3 receptor; NF-κB, nuclear factor-kappa B; OCT1, organic cationic transporter 1; PKA, protein kinase A; PKC, protein kinase C.

Several clinical studies have reported that the combination of LABA and LAMA reduces exacerbations more than the use of LABA or LAMA alone (Wedzicha et al., 2013; Calverley et al., 2018). LABA/LAMA combinations decrease hyperinflation (Beeh et al., 2014; Vincken et al., 2014) and symptom severity (Mahler et al., 2014; Buhl et al., 2015; Oba et al., 2016), and improve sputum clearance (Powrie et al., 2007; Meyer et al., 2011; Tagaya et al., 2016), all of which may contribute to decreasing exacerbations. Although the effects of the LABA/LAMA combination therapy against inflammation have not been demonstrated in patients with COPD, nor has LAMA or LABA alone, several mechanisms are possibly involved in the synergistic actions of the LABA/LAMA combination against inflammation (Figure 1). One of the possible mechanisms is that LABA and LAMA synergistically inhibit the release of non-neurogenic acetylcholine from bronchial epithelial cells. A recent report showed that, although single administration of glycopyrronium or indacaterol did not modify the release of acetylcholine from primary human bronchial epithelial cells, the combination of glycopyrronium and indacaterol significantly reduced the release of acetylcholine (Cazzola et al., 2016). This effect may originate through the modulation of organic cation transporter 1, a transporter for the release of acetylcholine from airway epithelial cells (Salomon et al., 2015). This mechanism may contribute to the anti-inflammatory effects by reducing the levels of acetylcholines, which have pro-inflammatory effects via M_3_ receptors. Another possible mechanism is that LABA and LAMA synergistically inhibit the release of pro-inflammatory factors from parenchymal cells or inflammatory cells. Tiotropium or olodaterol attenuates the production of reactive oxygen species (ROS), NOX-4 protein expression, and IL-8 release from 16HBE cells induced by cigarette smoke extract (CSE), and the combination of LABA and LAMA provides stronger effects compared to LAMA or LABA alone (Albano et al., 2018). Tiotropium or olodaterol reduces neutrophil adhesion and the expression of macrophage-1 antigen (MAC-1) adhesion protein on neutrophils stimulated by sputum supernatants from patients with COPD. The combination of these agents augments these effects. Olodaterol attenuates neutrophil accumulation and pro-inflammatory mediators in the airway of cigarette smoke or LPS-induced murine and guinea pig lung inflammation models, and also attenuates the release of TNF-α from blood leukocytes and reduces CD11b adhesion molecule expression on granulocytes *in vitro* (Wex et al., 2015). Recent studies showed that β2-receptor agonists attenuated the release of TNF-α and MCP-1 induced by LPS from a mouse macrophage cell line by mediating the cAMP-dependent inhibition of ERK and p38MAPK pathways (Keranen et al., 2016, 2017). Because a M_3_ mAChR antagonist attenuated the production of TNF-α induced by LPS from alveolar macrophages via mediating the NF-κB signaling pathway, combinations of these drugs may possibly inhibit the release of pro-inflammatory factors synergistically also in terms of intracellular signaling (Xu et al., 2012). To confirm these suggested mechanisms in the synergistic effects by the LABA/LAMA combination in COPD patients, further clinical investigations will be needed.

## Anti-Inflammatory Effects of ACh Via the α7 Nicotinic Acetylcholine Receptor (α7nAchR)

In contrast to the pro-inflammatory effects by ACh through muscarinic ACh receptors, it has been elucidated that there is a cholinergic anti-inflammatory pathway which includes parasympathetic nerves that regulate the inflammatory responses to maintain the body during tissue injury and infection. An investigation with gene-deficient animal models revealed that α7nAChR on splenic macrophages was critical for attenuating the inflammatory response by ACh in an LPS-induced animal septic model (Wang et al., 2003). Further investigations revealed that the efferent vagus nerve stimulates sympathetic neurons of the splenic nerve, and then NE produced by the splenic nerve stimulates splenic T cells expressing β2-adrenergic receptors causing the production of ACh, which can bind to α7nAChR on splenic macrophages (Rosas-Ballina et al., 2008; Rosas-Ballina et al., 2011; Vida et al., 2011).

In airways, ACh is derived from parasympathetic postganglionic neurons or non-neuronal cells including epithelial cells and can possibly bind to α7nAChR on macrophages and other inflammatory cells attenuating inflammation. An experiment using a mouse model of acid-induced acute lung injury or sepsis-induced acute lung injury showed that α7nAChR is expressed on alveolar macrophages and neutrophils, and activation of α7nAChR by nicotine or PNU-282987, a specific agonist, reduced the inflammatory responses and attenuated the severity of lung injury, whereas the lung injury became worse in α7nAChR-deficient mice (Su et al., 2007, 2010). In a rodent lung injury model of ventilator-induced lung injury, intestinal ischemia-reperfusion, severe acute pancreatitis and cardiopulmonary bypass-induced lung ischemia-reperfusion, the activation of α7nAChR provided protective effects against lung injury (Dos Santos et al., 2011; He et al., 2016; Ma et al., 2016; Ge et al., 2017). The exact mechanism by which the activation of α7nAChR induced anti-inflammatory effects is still not clear. The activation of a7 nAChR by a specific agonist in monocytes epigenetically increases histone deacetylation, which decreases in the NF-κB p65 activity of transcription factors including NF-κB, and the transcription of pro-inflammatory cytokine genes (Yang et al., 2017). In *E. coli* and LPS-induced lung injury mouse models, vagotomy augmented the lung recruitment of α7nAChR^+^ CD11b^+^ monocytes and neutrophils. The activation of α7nAChR by an agonist suppressed the recruitment of α7nAChR^+^ CD11b^+^ monocytes and neutrophils. The phosphorylation of serine 473 of AKT1 via α7nAChR signaling is involved in the attenuation of the lung recruitment of these inflammatory cells (Zhao et al., 2017). It has been also reported that activation of α7nAChR attenuates the polarization of alveolar macrophages to the pro-inflammatory M1 phenotype, whereas M2 macrophages were increased in an LPS-induced lung injury model (Pinheiro et al., 2017; Wang et al., 2018).

In contrast to acute lung injury models showing the protective effect of α7nAChR against inflammation, there is a report suggesting that signaling via α7nAChR may worsen fibrosis in the lungs. In a mouse bleomycin-induced lung fibrosis model, the severity of lung fibrosis, including histological scoring and the expression of fibrogenic genes, was attenuated in α7nAChR deficient mice. The activation of α7nAChR by GTS-21 (an α7nAChR agonist) increased the TGF-β-induced phosphorylation of Smad2/3 and transcription of fibrogenic genes in lung fibroblasts (Sun et al., 2017). Another report showed that nicotine stimulated collagen type I mRNA and protein expression in primary lung fibroblasts. This effect of nicotine was not observed in α7nAChR-deficient primary lung fibroblasts. *In vivo* administration of nicotine increased collagen type I expression in the wild type, but not in α7nAChR-deficient mice (Vicary et al., 2017). These reports suggest that α7nAChR signaling in lung fibroblasts is involved in the pathogenesis of fibrotic lung diseases and airway remodeling.

It has been reported that airway epithelial cells also express α7nAChR (Su et al., 2007; Gahring et al., 2017). Reporter gene analyses for α7nAChR expression in genetically modified mice revealed that α7nAChRs are expressed in club cells and alveolar type II cells as well as alveolar macrophages (Gahring et al., 2017). α7nAChRs are likely involved in the regulation of proliferation, ion transport and responses to inflammatory stimuli (Nastrucci and Russo, 2012; Maouche et al., 2013; Gahring et al., 2017). Moreover, α7nAChRs are expressed in non-small cell lung cancer cells and participate in tumor growth and epidermal-mesenchymal transition (Chernyavsky et al., 2015; Schaal and Chellappan, 2016; Bordas et al., 2017; Zhang et al., 2017; Mucchietto et al., 2018). Therefore, these tumor-promoting actions as well as pro-fibrotic actions via α7nAChRs could be serious barriers to employing α7nAChR agonists to attenuate inflammation in chronic inflammatory diseases such as COPD and asthma, which require continuous drug administration.

## Recent Advances in Clarifying the Neuroimmune Interactions Regulating Innate Lymphoid Cells

**FIGURE 2:**
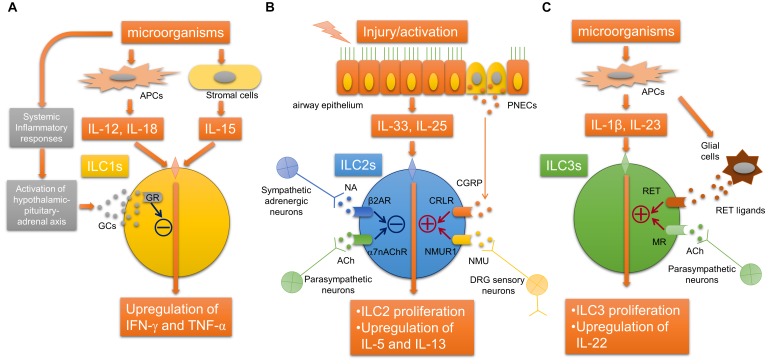
Neuroimmune interaction regulating innate lymphoid cells. **(A)** Glucocorticoids derived from hypothalamic-pituitary-adrenal axis stimulated by inflammatory mediators during systemic inflammation bind to GRs in ILC1s, which inhibits the release of IFN-γ. **(B)** The activation of ILC2s by epithelial alarmin cytokines is inhibited by ACh derived from parasympathetic neurons and possibly non-neuronal cells via α7nAChR, or NA derived from sympathetic neurons via β_2_AR. In contrast, neuromedin U derived from DRG sensory neurons or CGRP derived from PNECs augments the activation of ILC2s via NMUR1 or CRLR, respectively. **(C)** RET ligand derived from glial cells or acetylcholine derived from parasympathetic neurons arguments the proliferation and activation ILC3s via RET or muscarinic receptors, respectively. α7nAChR, α7 nicotinic acetylcholine receptor; ACh, acetylcholine; APC, antigen-presenting cell; β_2_AR, beta-2 adrenergic receptor; CGRP, calcitonin gene-related peptide; CRLR, calcitonin receptor-like receptor; DRG, dorsal root ganglia; GCs, glucocorticoids; IFN-γ, interferon-gamma; ILCs, innate lymphoid cells; MR, acetylcholine muscarinic receptor; NA, norepinephrine; NMU, neuromedin U; NMUR1, neuromedin-U receptor 1; PNEC, pulmonary neuroendocrine cell; TNF-α, tumor necrosis factor-alpha.

It has been revealed that ILCs, which originate from common lymphoid cells but do not have antigen receptors, exist in various tissues of mammals including human, and can produce various cytokines in response to various stimuli including alarmins. ILCs are classified into type 1, type 2, and type 3 ILCs according to their functions and the cytokines produced (Vivier et al., 2018). Recently, neurotransmitters including acetylcholine have been shown to likely participate in the regulation of proliferation and activation of ILCs (Figure 2). Type 2 innate lymphoid cells (ILC2s) produce significant amounts of Th2 cytokines and participate in airway allergic inflammation. A recent report described that α7nAChRs are expressed on ILC2s. The activation of α7nAChRs on ILC2s suppresses IL-5 and IL-13 production in ILC2s and ameliorates ILC2-induced airway hyperreactivity. α7nAChR agonist attenuates the expression of GATA3 and NF-κB in ILC2s as well as the proliferation of ILC2s. The activation of α7nAChR by an agonist also prevents human ILC2 mediated airway hyperreactivity as well as type 2 cytokine production and eosinophilic inflammation. These observations may suggest a protective role of α7nAChR signaling in ILC2s against ILC2-mediated allergic inflammation and the possibility that α7nAChR could be a potential therapeutic target for the treatment of ILC2-mediated asthma.

Other recent studies have shown that the neuropeptide neuromedin U (NMU) released from neural cells activates ILC2s and amplifies ILC2-driven allergic inflammation (Cardoso et al., 2017; Klose et al., 2017; Wallrapp et al., 2017). In an airway allergic mouse model induced by intranasal administration of IL-25 and IL-33, single-cell RNA sequencing analyses were performed to profile mouse lung-resident ILCs and revealed that NMUR1, a receptor for NMU, was specifically expressed on ILC2s (Wallrapp et al., 2017). Immunofluorescence microscopic analysis suggested that ILC2s were likely located close to nerve fibers. Sensory neurons in the dorsal root ganglia, but not parasympathetic neurons in the nodose/jugular ganglion, expressed NMU. IL-13 induced the upregulation of NMU in cultured dorsal root ganglia neurons. NMU can activate ILC2s and induce type 2 cytokine productions *in vitro*. Intranasal co-administration of NMU with IL-25 strongly amplified airway allergic inflammation. Moreover, NMUR1-deficient mice showed an attenuation of both the ILC2 frequency and effector function during airway inflammation from house dust mites. These findings indicated that NMU derived from sensory neurons innervated into airways can activate ILC2s, and this newly identified neuroimmune interaction is involved in the modulation of airway allergic inflammation (Wallrapp et al., 2017).

Another study using RNA sequencing analyses of ILC2s revealed that mouse and human ILC2s express β_2_-adrenergic receptor (β_2_AR) (Moriyama et al., 2018). β_2_AR-deficient mice showed amplified ILC2 activation and airway allergic inflammation induced by intranasal stimulation with IL-33 or *Alternaria* extract, whereas the administration of β_2_ agonist attenuated the ILC2-responses and allergic inflammation. RNA-sequence analyses further showed that β_2_AR signaling pathway inhibits the cell proliferation and effector function of ILC2s. This study suggests that sympathetic adrenergic neurons that innervate the airways negatively regulate ILC2s via β_2_AR.

Pulmonary neuroendocrine cells (PNECs) are a unique but poorly understood cell population in the lungs. Although it has been reported that PNECs are increased in pulmonary diseases, including small-cell lung cancer, the precise role of PNECs in pulmonary diseases remains unknown. A recent report showed that PNECs have a significant role in the mucosal type 2 response in models of allergic asthma (Sui et al., 2018). PNECs act through calcitonin gene-related peptide (CGRP) to stimulate ILC2s and induce downstream immune responses, and also act through GABA to induce goblet-cell hyperplasia. These new findings demonstrated a novel neuroimmune system in the airway that augments the type 2 allergic response. It is still unknown how PNECs are activated following allergen challenge. PNECs are innervated by sensory afferents mostly from nodose ganglion neurons and vagal efferents, and this innervation of PNEC is required for the increased production of GABA (Barrios et al., 2017), suggesting that stimulating efferent neurons that innervate PNECs may be involved in the activation of PNECs.

Compared with ILC2s, neuroimmune interactions that regulate the other ILCs, ILC3 and ILC1, in airways remain unclear, although it has been reported that their possible interactions may regulate other ILCs during systemic inflammation or inflammation of other organs. In gut, ILC3s express RET, a neuroregulatory receptor, and are localized close to glial cells expressing RET-ligands. Glial cells sense microenvironmental changes and release RET-ligands, causing ILC3 activation and the release of IL-22 (Ibiza et al., 2016). In a mouse *E. coli* peritonitis model, acetylcholine, probably via muscarinic receptors, upregulates ILC3 numbers, causing peritoneal macrophage production of a protective mediator, protectin conjugates in tissue regeneration 1 (PCTR1), and accelerate the resolution of inflammation induced by bacterial infection (Dalli et al., 2017). In a study using an LPS-induced septic model, group 1 ILCs expressed glucocorticoid receptor and stimulation with glucocorticoids prevented IFN-γ production by ILC1s, which cause the development of IL-10-dependent tolerance to endotoxin (Quatrini et al., 2017).

These on-going investigations of neuroimmune crosstalk, including the cholinergic pathways in airways, may lead to new potential therapeutic targets not only for acute inflammation but also for chronic inflammatory respiratory diseases including COPD. However, we should also note that neurotransmitters participating in neuroimmune interactions also have other fundamental functions including neurotransmission, which means blocking these neurotransmitters could possibly cause critical adverse effects. Accordingly, we also have to take into account the delivery system for candidate drugs that regulate neuroimmune interactions.

## Conclusion

Accumulating evidence from *in vitro* and *in vivo* animal model investigations suggests that acetylcholine derived from parasympathetic neurons and non-neuronal cells can modulate airway inflammation. The pro-inflammatory actions of acetylcholine, mainly via M_3_ receptors, and possible synergistic anti-inflammatory effects of β_2_AR signaling may provide evidence for the benefit of treatment with LAMA or the combination of LABA/LAMA, although it remains unclear whether these treatments really have anti-inflammatory effects in COPD patients. Therefore, further clinical investigation will be needed to clarify the anti-inflammatory effects. Anti-inflammatory effects by acetylcholine via α7nAChR, which may modulate lung inflammation, have been revealed especially in systemic inflammation. However, we should be careful about other biological effects of α7nAChR signaling, including pro-fibrotic actions and tumor-promoting actions in terms of therapeutic utilization for chronic respiratory diseases including COPD. Recent on-going studies has revealed critical interactions between ILCs and neurotransmitters including acetylcholine. These newly found neuroimmune interactions may reveal therapeutic targets for airway inflammatory diseases. Further investigations will reveal novel effects of neurotransmitters including acetylcholine on immune systems and inflammation and may provide new therapeutic clues for clarifying airway inflammatory diseases such as COPD.

## Author Contributions

All authors listed have made a substantial, direct and intellectual contribution to the work, and approved it for publication.

## Conflict of Interest Statement

The authors declare that the research was conducted in the absence of any commercial or financial relationships that could be construed as a potential conflict of interest.
